# Rapid detection of *Clostridium perfringens* in food by loop-mediated isothermal amplification combined with a lateral flow biosensor

**DOI:** 10.1371/journal.pone.0245144

**Published:** 2021-01-07

**Authors:** Thanawat Sridapan, Wanida Tangkawsakul, Tavan Janvilisri, Wansika Kiatpathomchai, Sirintip Dangtip, Natharin Ngamwongsatit, Duangjai Nacapricha, Puey Ounjai, Surang Chankhamhaengdecha

**Affiliations:** 1 Graduate Program in Molecular Medicine, Faculty of Science, Mahidol University, Bangkok, Thailand; 2 Center of Nanoscience and Nanotechnology, Faculty of Science, Mahidol University, Bangkok, Thailand; 3 Department of Biochemistry, Faculty of Science, Mahidol University, Bangkok, Thailand; 4 Bioengineering and Sensing Technology Research Team, National Center for Genetic Engineering and Biotechnology (BIOTEC), National Science and Technology Development Agency (NSTDA), Pathum Thani, Thailand; 5 Department of Clinical Sciences and Public Health, Faculty of Veterinary Science, Mahidol University, Nakhon Pathom, Thailand; 6 Department of Chemistry, Faculty of Science, Mahidol University, Bangkok, Thailand; 7 Department of Biology, Faculty of Science, Mahidol University, Bangkok, Thailand; Cornell University, UNITED STATES

## Abstract

*Clostridium perfringens* is a key anaerobic pathogen causing food poisoning. Definitive detection by standard culture method is time-consuming and labor intensive. Current rapid commercial test kits are prohibitively expensive. It is thus necessary to develop rapid and cost-effective detection tool. Here, loop-mediated isothermal amplification (LAMP) in combination with a lateral-flow biosensor (LFB) was developed for visual inspection of *C*. *perfringens*-specific *cpa* gene. The specificity of the developed test was evaluated against 40 *C*. *perfringens* and 35 other bacterial strains, which showed no cross-reactivity, indicating 100% inclusivity and exclusivity. LAMP-LFB detection limit for artificially contaminated samples after enrichment for 16 h was 1–10 CFU/g sample, which was comparable to the commercial real-time PCR kit. The detection performance of LAMP-LFB was also compared to culture-based method using 95 food samples, which revealed the sensitivity (SE), specificity (SP) and Cohen's kappa coefficient (κ) of 88.0% (95% CI, 75.6%-95.4%), 95.5% (95% CI, 84.8%-99.4%) and 0.832 (95% CI, 0.721–0.943), respectively. Area under the receiver operating characteristic (ROC) curve was 0.918 (95% CI, 0.854–0.981), indicating LAMP-LFB as high relative accuracy test. In conclusion, LAMP-LFB assay is a low-cost qualitative method and easily available for routine detection of *C*. *perfringens* in food samples, which could serve as an alternative to commercial test kit.

## Introduction

*Clostridium perfringens* is as an anaerobe responsible for a wide range of diseases such as gas gangrene and food poisoning in human, necrotic enteritis in animals worldwide [1]. It is one of the most common pathogens that causes foodborne illness in the United States. According to the Centers for Disease Control and Prevention (CDC), an estimate of nearly one million cases of food poisoning occurs each year [2]. The pathogenicity of *C*. *perfringens* based on production of four major toxins (alpha, beta, epsilon and iota), which have been used to classify *C*. *perfringens* into five distinct types (A-E) [1]. Type A strains are predominantly involved in food poisoning outbreaks, which produce alpha toxin, encoded by the *cpa* gene as well as concomitantly produce the enterotoxin (CPE) in the digestive tract responsible for gastroenteritis and diarrhea [3].

*C*. *perfringens* type A food borne illness primarily involves high-protein foods like raw meat including poultry meat that have not been cooked thoroughly [4]. Other food vehicles such as gravies and chili paste have also been frequently reported [2, 5]. Thus, detection of *C*. *perfringens* in food samples is required to ensure food safety for consumers as well as to avoid product recalls for manufacturers. Culture-based assay is the gold standard method for this pathogen. Although, the microbial culture-based detection is reliable and inexpensive, however it is laborious and time-consuming, which requires at least 3 days for completion [6]. At present, rapid diagnostic tests based on molecular detection such as real-time PCR have been commercially available for detection of foodborne pathogens including *C*. *perfringens* [7]. Such detection kit represents qualitative detection of a highly conserved DNA sequence specific to the *cpa* gene, which is presented in all *C*. *perfringens* strains [8–10]. However, it is rather expensive and requires costly instrumentation, therefore may not be suitable for the point of care detection and resource-limiting settings. Thus, it is of interest to develop a rapid detection tool, which is cost-effective, sensitive, labor-saving and exhibits immediate results as an alternative to the current methods.

Loop-mediated isothermal amplification (LAMP) has been widely employed in various fields [11]. This assay is extremely sensitive and requires much shorter time to obtain results compared to the conventional culture and PCR methods [8, 9, 12–14]. Additionally, various detection formats such as turbidity, colorimetric detection, gel electrophoresis and lateral flow biosensor (LFB) can be used to detect LAMP products [15], rendering LAMP ideal for application under point-of-care and resource-limited scenarios. LFB has gained popularity as it can be applied for visualization of LAMP products with rapid, easy and no requirement for any equipment [16]. Recently, the LAMP combined with LFB (LAMP-LFB) assay has been reported for the detection of foodborne pathogens for example *Escherichia coli* [17], *Listeria* spp. [18], *Shigella* spp. [19], *Staphylococcus aureus* [20, 21], *Vibrio cholera* [22], *Vibrio parahaemolyticus* [23] and *Vibrio vulnificus* [24]. To date, LAMP assay targeting *C*. *perfringens cpa* gene [8, 9, 25], has been reported with the visualization by turbidity, agarose gel electrophoresis and colorimetric detection. However, LAMP-LFB has not been demonstrated for the detection of *C*. *perfringens*.

Therefore, in this study we designed LAMP primer sets and DNA probe to amplify the *cpa*-specific target of *C*. *perfringens* combined with LFB for visual detection of the LAMP products. The LOD and detection performance of the developed LAMP-LFB for *C*. *perfringens* detection were compared to commercially available real-time PCR kit and culture-based method, respectively.

## Materials and methods

### Bacterial strains

A total of 75 bacterial strains were included in this study (S1 Table). These strains were cultured according to the requirements of individual organisms. *C*. *perfringens* DMST 16637, which was used as the standard strain to develop LAMP-LFB assay, was cultivated overnight at 37°C on sheep blood agar (Clinical Diagnostic, Thailand) in an anaerobic condition with the AnaeroPack system (Mitsubishi Gas Chemical Co., Inc., japan). One colony was suspended into 5 mL of brain heart infusion (BHI) broth (Himedia, India), then anaerobically incubated overnight at 37°C for DNA extraction. *Campylobacter* spp. and *Streptococcus* spp. were cultured at 37°C on sheep blood agar in a microaerophilic condition created with the atmosphere generation systems (CampyGen and CO_2_Gens, Oxoid, USA), respectively. Other bacterial strains were grown in nutrient-rich medium, Luria Bertani Broth (LB) and BHI (Himedia) by placing in shaking incubator at 37°C for 16 h.

### DNA extraction

Boiling method was applied to extract DNA from pure culture and food sample as previously described with some modifications [8, 9, 26]. Briefly, homogenate was centrifuged at 90 × *g* for 3 min at room temperature (RT). The supernatant (⁓ 400 μl) was transferred in a new microtube and centrifuged to be concentrated at 8,000 × *g* for 5 min at RT. The supernatant was discarded and resuspended in 500 μl of 1× phosphate buffer saline, pH 7.2. Next, the mixture was centrifuged at 8,000 × *g* for 5 min at RT, and the supernatant was discarded. The cell pellets were resuspended in 100 μl of 1× Tris EDTA buffer, pH 8.0, boiled for 20 min, and immediately placed on ice for 5 min. Finally, a centrifugation at 14,000 × *g* for 10 min at RT was performed to pellet cell debris and the supernatant DNA was transferred to a new microtube and stored at -20°C until use. DNA samples were quantified using the NanoDrop spectrophotometer (Biochrom., USA).

### LAMP primers and detection probe

A set of four LAMP primers including F3, B3, FIP and BIP were designed using Primer Explorer V5 software program (http://primerexplorer.jp/lampv5e/index.html) based on *C*. *perfringens* phospholipase C, *cpa* gene (GenBank accession number NC_008261.1) (Table 1). A poly T linker was added in the FIP and BIP between F1c - F2 and B1c - B2. For *C*. *perfringens* detection by LAMP-LFB assay, FIP was modified with digoxigenin at the 5’ end. The region between the F1c and B1c primer targets was selected to design DNA probe modified with fluorescein iso-thiocyanate (FITC) at 5’ end. All oligonucleotides were synthesized by Bio Basic Inc., Canada.

**Table 1:** Primers and probes for LAMP-LFB detect of *C*. *perfringens*.

Primer	Length	Sequence (5'-3')
F3	22	TCTGGGATCCTGATACAGATAA
B3	25	CTATATCTCCAAAATAGTGCATAGC
^a^FIP	47	TGTGATTCCCCTGTGTCAGG-TTTT-TTTCTCAAAGGATAATAGTTGGT
BIP	49	GCATTAGCTAGATATGAATGGC-TTTT-CCAAGATAGAATGTAGCTTGTT
^b^DNA-Probe	16	AATAAGAAAATTTTCA

^**a**^ 5’-modified with digoxigenin.

^**b**^ 5’-modified with fluorescein isothiocyanate (FITC).

### LAMP-LFB assay conditions

The LAMP reaction mixture (25 μl) consisted of 1× ThermoPol® Reaction buffer, 6 mM MgSO_4_, 1.4 mM of dNTPs (Vivantis, Malaysia), 0.2 μM F3 and B3 primers, 1.6 μM FIP and BIP primers, 0.4 M betaine (Sigma, USA), 8 U of *Bst* DNA polymerase, large fragment (New England Biolabs Inc., USA) and 2 μl of template DNA. The reaction was performed at 62°C for 40 min. The LAMP products were then hybridized with FITC-modified DNA probe (0.2 μM) at 62°C for 5 min and heat inactivated at 85°C for 5 min. For visualization of LAMP-LFB results, 0.5 μl of hybridized LAMP products (digoxigenin-labeled LAMP amplicon and FITC-labeled probe) were added into the microtube containing 120 μl of running buffer (PBS and Surfynol® 465 surfactant) (Kestrel Bio Sciences, Thailand). All mixture volume was added into the sample pad of LFB strip (Kestrel Bio Sciences, Thailand) and waited for 2 min. A red color line must be visualized at the quality control line (C line) of the LFB strip to confirm that the test was correctly operated (S1 Fig). The obtained hybridized LAMP products were also subjected to electrophoresis on a 1.5% agarose gel, with Serva DNA stain G staining (SERVA Electrophoresis GmbH, Germany), and visualized using a UV transilluminator (Syngene, USA).

### Specificity of LAMP-LFB assay

The specificity of the developed LAMP-LFB assay was examined under the condition described above using 20 ng each of template DNA of the 75 bacterial strains (S1 Table) including 40 *C*. *perfringens* and 35 other strains. The distilled water was used as a reagent blank control.

### Determination of the limit of detection (LOD) in pure culture and in artificially inoculated food samples

To estimate the sensitivity of the LAMP-LFB assay, template DNA from *C*. *perfringens* DMST 16637 was quantified at 260 nm using the NanoDrop spectrophotometer, then was 10-fold serially diluted to concentrations ranging from 100 ng/μl to 1 fg/μl. Additionally, the turbidity of an overnight culture of *C*. *perfringens* in BHI broth was adjusted to 0.5 McFarland (Grant Bio™ Densitometer, UK), which corresponded to 10^6^ colony forming units (CFU)/mL as determined experimentally. Then, ten-fold serial dilutions were prepared in 0.85% saline solution (NaCl) to concentrations ranging from 0.01−10^6^ CFU/mL. One milliliter of each dilution was collected for genomic DNA extraction using boiling method as described above to determine the detection limit in pure culture. Meanwhile, 1 mL of each dilution was pipetted on solidified tryptose sulfite cycloserine (TSC) agar (TSC; M837I, ISO 7937:1985) (Himedia, India) without egg yolk, then 15 mL of TSC agar were additional poured into the dish, mixed by gently swiveling dish. The assay was conducted in two replicates and incubated at 37°C for 16 h in an anaerobic condition. Black colonies on the agar plate were counted and used to calculate the number of CFU in the *C*. *perfringens* suspension.

To determine the utility of the LAMP-LFB assay, ten grams of each food matrix including, chili paste, cured meat and gravy sauce were weighted and aseptically collected to the stomacher bag (BagPage Plus 400, Interscience, France) containing 90 mL of fluid thioglycolate (FTG) medium (Himedia, India), then homogenized for 30 s at low speed (BagMixer 400 Lab Blender, Interscience, France). The aliquot of 100 μl of each ten-fold serial dilution of *C*. *perfringens* as described above was spiked into the food homogenate. Moreover, 100 μl of 0.85% NaCl was added to the sample as a control for the absence of *C*. *perfringens* in the original food materials. All homogenate samples were enriched at 37°C in anaerobic condition for 0, 3, 6, 10, 12, 16, 18 and 24 h. Then, 1 mL of enriched food samples of each dilution was collected for genomic DNA extraction using boiling method. The analytical sensitivity test was conducted in two replicates, and the last dilution in each spiked sample that test positive was considered as the LOD.

A comparative analysis of the LAMP-LFB and the commercial real-time PCR kit (SureFast® *Clostridium perfringens* PLUS, Germany) was conducted in this study. The commercial real-time PCR mixture contained 20 μl of reaction mix with *Taq* polymerase and 5 μl of genomic DNA, which was prepared as described earlier. The reactions were performed and analyzed by using an Applied Biosystems 7500 real-time PCR machine with an initial denaturation at 95°C for 1 min and 45 cycles of denaturation at 95°C for 15 s, annealing and extension at 60°C for 30 s. The positive reaction was defined as a sample DNA showed amplification in the FAM-channel, while the negative reaction showed no amplification in the FAM-channel according to instruction manual.

### Performance evaluation of the LAMP-LFB in food samples

A total of 95 food samples including chili pastes (n = 70), cured meats (n = 20) and gravy sauces (n = 5) were collected between July and September 2019 from the markets including fresh markets (n = 45) and supermarkets (n = 50) in Bangkok and vicinity, Thailand (S2 Table). The samples were kept in sterile containers and analyzed immediately for detection of *C*. *perfringens*. Ten grams of each sample were homogenized in 90 mL FTG (Himedia, India) in a stomacher bag (BagPage Plus 400, Interscience, France) for 30 s at low speed and then incubated at 37°C for 16 h in anaerobic conditions. Following incubation, 1 mL of enrichment medium was collected for genomic DNA extraction using boiling method, and 1 mL of each enriched culture was pipetted onto TSC and poured with TSC agar as described above. Considering culture-based method as the gold standard, the sensitivity (SE) and specificity (SP) of LAMP-LFB assay were calculated with 95% confidence intervals (95% CIs) using MedCalc (https://www.medcalc.org/calc/diagnostic_test.php). The degree of agreement between the result from different test was determined using Cohen's kappa coefficient (κ) (https://www.graphpad.com/quickcalcs/kappa1/). In addition, a receiver operating characteristic (ROC) analysis was plotted to evaluate the detection accuracy of the tests using SPSS software-version 18.0.

## Results

### Specificity of LAMP-LFB assay

**Fig 1:**
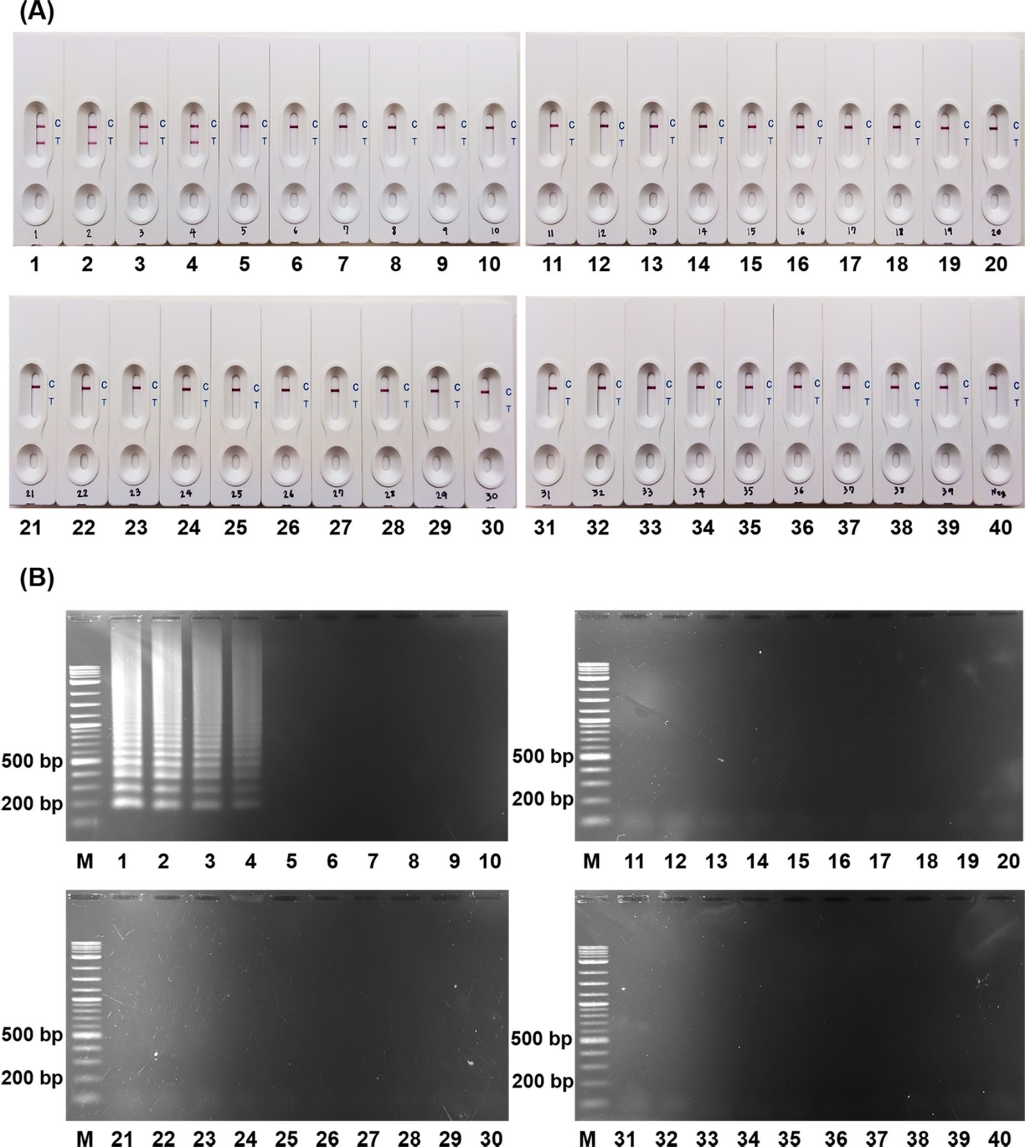
The specificity of LAMP assay for *C*. *perfringens*. LAMP products using 20 ng of DNA templates from different bacterial strains as detected by (A) LFB in comparison to (B) 1.5% agarose gel electrophoresis. C indicates the control line and T indicates the test line. A positive result displays bands at both C and T line, while a negative result shows one band at the C line. M: 2-log DNA ladder. 1–4: *C*. *perfringens* (DMST 16637, ATCC 3626, Ue2130/06, NCTC 8084); 5–7: *C*. *botulinum* (NCTC 0727, NCTC 0751, NCTC 11219); 8–10: *C*. *difficile* (NIH 2, R20291, H37 [27]); 11: *B*. *cereus* ATCC 14579; 12: *C*. *coli* DMST 18034; 13: *C*. *jejuni* DMST 15190; 14: *E*. *coli* DMST 703; 15: *L*. *monocytogenes* DMST 17303; 16: *S*. Abony DMST 21863; 17: *S*. Bangkok DMST 7121; 18: *S*. Derby DMST 8535; 19: *S*. Enteritidis DMST 15676; 20: *S*. Hvittingfoss DMST 15681; 21: *S*. Paratyphi B DMST 28118; 22: *S*. Senftenberg DMST 17013; 23: *S*. Typhi DMST 22842; 24: *S*. Typhimurium ATCC 23566; 25: *S*. Wandsworth DMST 19204; 26: *S*. Waycross DMST 19205; 27: *S*. *boydii* DMST 30245; 28–30: *S*. *aureus* (ATCC 25923, DMST 8013, DMST 4745); 31: *S*. *epidermidis* DMST 15505; 32: *S*. *haemolyticus* DMST 15511; 33: *S*. *sacharolyticus* DMST 15512; 34: *S*. *pyogenes* DMST 4369; 35: *S*. *pneumonia* DMST 7945; 36: *S*. *suis* DMST 18783; 37: *V*. *cholera* DMST 2873; 38: *V*. *vulnificus* DMST 21245; 39: *Y*. *enterocolitica* DMST 8012; 40: Blank control.

To determine the specificity of LAMP primer set, the LAMP reactions were carried out with 40 *C*. *perfringens* and 35 other bacterial strains (S1 Table). LAMP products were visualized by LFB and gel electrophoresis. A positive result as defined by the development of visual lines at both the test line (T line) and control line (C line) of a LFB (Figs 1A and S2A) and ladder-like bands on gel electrophoresis (Figs 1B and S2B) were detected in all *C*. *perfringens* strains. Only a control line on the LFB and no ladder-like patterns were observed in other bacterial strains and in the blank control. These results indicated that the designed LAMP primers were highly specific to the *C*. *perfringens* with 100% inclusivity and 100% exclusivity. In addition, the positive results of LAMP-LFB were in accordance with those obtained from the gel electrophoresis.

### LOD of the LAMP-LFB assay

**Fig 2:**
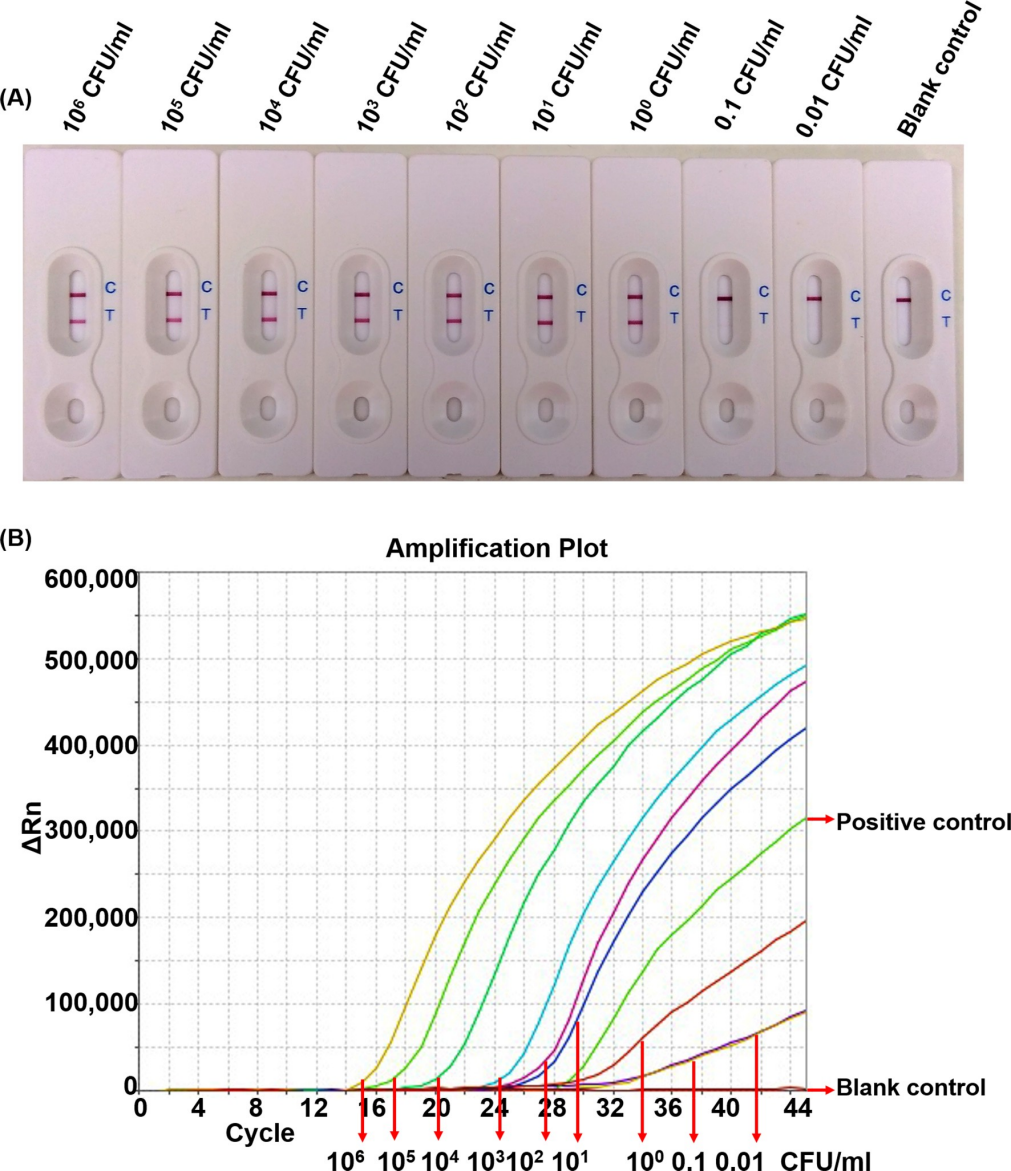
The LOD of LAMP-LFB (A) and commercial real-time PCR kit (B) for detecting *C*. *perfringens* using serial tenfold dilutions of DNA extracted in pure culture (from 10^6^−0.01 CFU/mL). Positive control of commercial real-time PCR kit showed amplification in the FAM channel, while negative control showed no amplification.

To determine the LOD of the LAMP-LFB assay in pure culture, genomic DNA extracted from serial dilutions of *C*. *perfringens* strain DMST 16637 culture were used as templates. The results showed that the LAMP-LFB assay detected as low as 10 pg/μl of genomic DNA (S3 Fig) or 1 CFU/mL of pure culture (Fig 2A), while the commercialized real-time PCR kit has the LOD of ≤ 5 DNA copies (18 fg) according to manufacturer’s manual or 0.01 CFU/mL of pure culture (Fig 2B).

**Fig 3:**
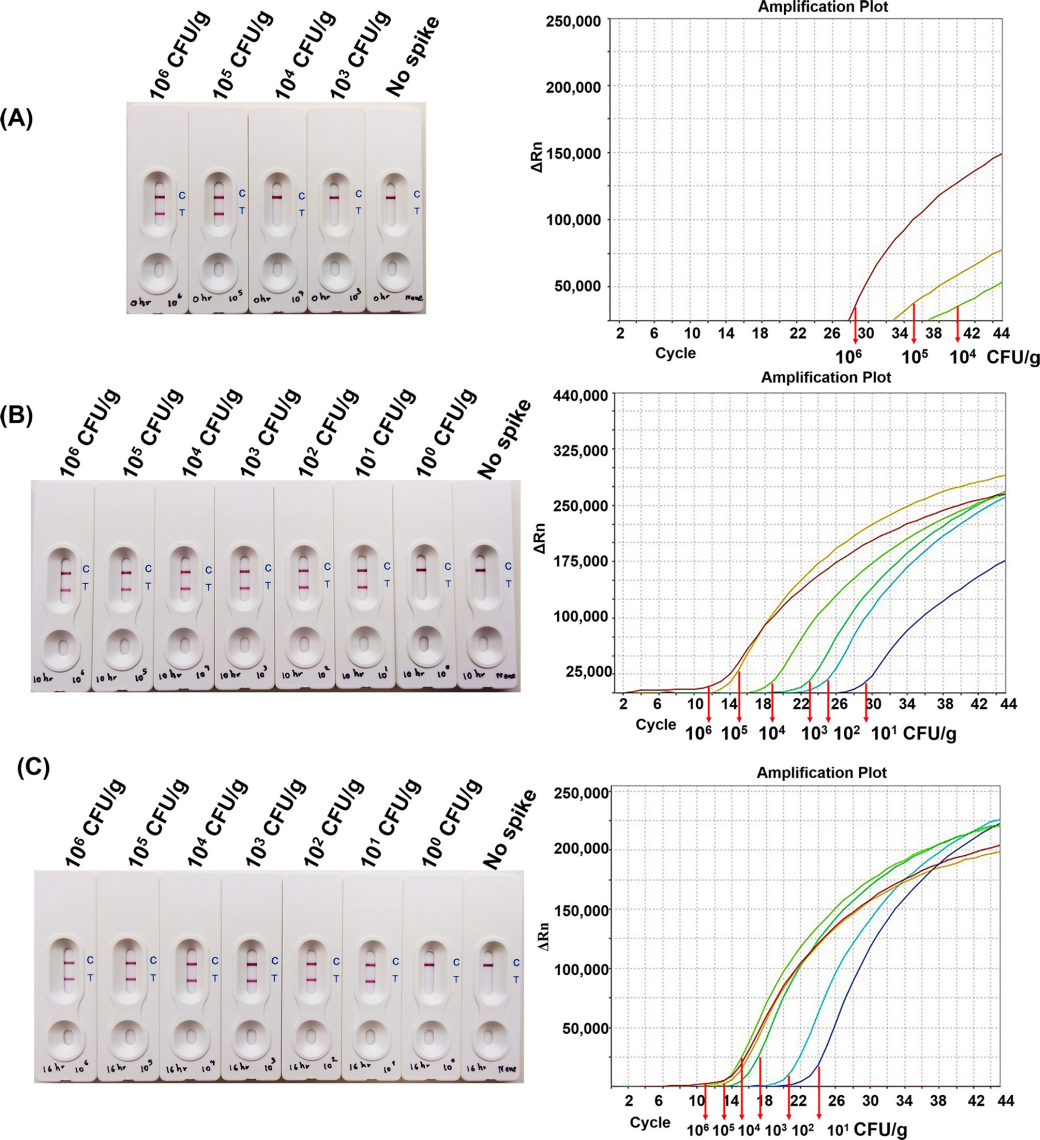
The LOD of LAMP-LFB and commercial real-time PCR kit using chili paste spiked with *C*. *perfringens*. The LOD was compared between LAMP-LFB (left) and commercial real-time PCR kit (right), enriched at 37°C for 0 h (A), 10 h (B) and 16 h (C).

The practical application of the LAMP-LFB assay was further explored by evaluating the LOD of *C*. *perfringens* in artificially contaminating food samples. Without the enrichment, the LOD of LAMP-LFB and commercial real-time PCR kit assays were 10^5^ and 10^4^ CFU/g, respectively (Fig 3A). When spiked food was enriched, the LOD of incubation time at 3 and 6 h was similar to non-enrichment. Upon the enrichment for 10 to 24 h, the LOD levels of the LAMP-LFB and commercial real-time PCR were both improved and comparable at 10 CFU/g. (Fig 3B and 3C). No positive results were observed from non-spiked samples. These results indicated that the enrichment culture step is required to enhance the target *C*. *perfringens* cell number in the enrichment broth prior to LAMP-LFB assay. Therefore, the 16 h incubation step was employed in further experiments to evaluate the LOD in other food matrices. Two spiked samples including gravy sauce and bologna were inoculated with different concentrations of *C*. *perfringens* ranging from 10^4^−10^6^ CFU/g for non-enrichment and 0.01−10^4^ CFU/g for enrichment at 16 h. The LOD of LAMP-LFB was equivalent to commercial real-time PCR kit at 16 h enrichment for both types of food samples (Table 2 and S4 and S5 Figs). These results indicated that *C*. *perfringens* in spiked food samples could be detected by two assays with the same LOD after the enrichment procedure.

**Table 2:** LOD of *C*. *perfringens* DMST 16637 in artificial food products by LAMP-LFB and commercial real-time PCR kit.

Type of sample	Methods	Limit of detection (CFU/g)			
		Spiked samples with enrichment period for:		Non-spiked samples with enrichment period for:	
		0 h	16 h	0 h	16 h
Chili paste	Real-time PCR	10^4^	10	-	-
	LAMP-LFB	10^5^	10	-	-
Gravy sauce	Real-time PCR	10^5^	1	-	-
	LAMP-LFB	10^5^	1	-	-
Bologna	Real-time PCR	10^4^	10	-	-
	LAMP-LFB	10^5^	10	-	-

### Evaluation of LAMP-LFB in naturally contaminated food sample

To evaluate the LAMP-LFB assay for its detection performance, of the 95 samples tested, 50 samples (52.6%) including 29 samples from fresh markets and 21 samples from supermarkets were found positive by culture-based method, while LAMP-LFB assay detected 44 positive samples (46.3%) (23 from fresh markets and 21 from supermarkets) with six as false-negative results, giving it a sensitivity of 88.0% (95% CI, 75.6%-95.4%). Of 45 samples indicated as negative by culture-based method, two were positive as false-positive results by LAMP-LFB assay, yielded a specificity of 95.5% (95% CI, 84.8%-99.4%) (S3 and S4 Tables). The Cohen kappa index of the LAMP-LFB assay was 0.832 (95% CI, 0.721–0.943), which had a very good agreement with the reference method (S4 Table). To determine the detection accuracy of LAMP-LFB assay, ROC analysis revealed that the curve was closest to the upper left corner with area under the curve of 0.918 (95% CI, 0.854–0.981), indicative of a high accuracy test (S6 Fig).

## Discussion

The novel LAMP-LFB assay was successfully developed to detect of *C*. *perfringens* in food samples. The primers and probe were designed specifically to target the *cpa* gene, which is present in all types of *C*. *perfringens*. The exclusivity with other 35 bacterial strains indicated a high specificity of the designed primers. In comparison to the commercially available real-time PCR kit, the detection limit of LAMP-LFB was in a similar range from 1–10 CFU/g in spiked food samples with the enrichment, making it feasible and cost-effective alternative for the detection of *C*. *perfringens*. In addition, this assay provides test results less than 24 h, significantly superior to standard culture-based method.

Based on the food safety of international criteria, the limit for *C*. *perfringens* in ready-to-eat food ranges from < 10 CFU/g [28], < 20 CFU/g [29], < 100 CFU/g [30, 31] and 100–1,000 CFU/g [32]. The LOD of LAMP-LFB in spiked sample was 10^4^−10^5^ CFU/g without enrichment step and with the enrichment step of at least 10 h the detectable level ranged 1–10 CFU/g for various food matrices. An incubation time at 10–24 h could gave the same LOD, so the incubation time for enrichment required more than 10 h, which appear to be sufficient for the detection in all spiked food samples by LAMP-LFB. However, in this study, the incubation time at 16 h for enriched samples was selected because the results can be obtained as soon as next day after sample receipt within two 8-h working shifts, including enrichment, DNA extraction and LAMP-LFB detection. Importantly, developed LAMP-LFB assay could detect low number of *C*. *perfringens* that could be present lower than the microbiological limits as aforementioned. Qualitative LAMP-LFB assay here indicates the potential use for rapid monitoring of *C*. *perfringens* contamination, which could be recalled from the food industry for preventing of health threats. The industries would take precautionary steps and improve the sanitary cleaning procedure in the processing facilities as well.

There have been reports on LAMP assay to detect *C*. *perfringens* in food samples, which revealed that the LOD either equal to real-time PCR [9] or more sensitive than that of conventional PCR [8, 9, 12]. In previous spiking studies without an enrichment step, the LOD of the developed LAMP-LFB assay is close to other reports, which revealed the LOD of 10^2^−10^4^ CFU/g [9] and 10^7^ CFU/g [8] based on targeting the *cpa* gene, 10^4^ to more than10^7^ CFU/g based on the *cpe* gene [12]. Our data agreed with those previous reports that LAMP assay required more than 10^3^ CFU/g to yield a sufficient amount of *C*. *perfringens* for detection [8, 12]. When spiked food samples were enriched for at least 6 h, the LOD of LAMP assay was improved [8, 12]. Meanwhile, a minimum enrichment time of our LAMP-LFB assay required more time of 10 h. However, the cost for in-house DNA preparation protocol in this study was reduced by using boiling method. In contrast, the commercial DNA preparation kits were applied in some reports [8, 12], which are costly, laborious and require a large number of reagents and equipment, resulting in the risk of carryover contamination [12].

In addition, the developed LAMP-LFB assay was comparable to that of commercial real-time PCR kit. LAMP-LFB has great advantage of over real-time PCR in term of simple and easy operation. At the same time, its ability to amplify DNA target under constant temperature allowing the use of easy and cost-effective instrument, while real-time PCR requires specialized equipment. Even though, the LOD of the developed assay was less sensitive than that of commercial real-time PCR kit both in pure culture and in spiking study without enrichment step culture, as LAMP-LFB was able to detect 10^5^ CFU/g, while commercial real-time PCR kit had the ability to detect 10^4^ CFU/g of chili paste and bologna (Figs 2 and 3A and S5A and Table 2). This can be explained by the fact that the interpretation of test results of both assay is different. Results of LAMP-LFB can be easily interpreted by visual observations for the presence or absence of the test and control line, while real-time PCR results require a fluorescence reader in a real-time PCR machine to measure the fluorescence signal and the interpretation of positive and negative results require the use of positive and negative controls given in the kit [33, 34]. However, the enrichment culture step of more than 10 h allows the LOD of LAMP-LFB to be comparable that of commercial real-time PCR kit with the range of 1–10 CFU/g. (Figs 3B and 3C and S4B and S5B and Table 2). Thus, an enrichment culture step is beneficial by improving the detection limit to be comparable to the commercial kits.

Importantly, previous studies reported the assays developed in spiking experiments, but a thorough evaluation in real food products was missing [9, 12]. The LAMP-LFB developed here showed prospective application of detection performance (88.0% sensitivity (95% CI, 75.6%-95.4%) and 95.5% specificity (95% CI, 84.8%-99.4%) for evaluation using 95 sample of food from fresh and supermarkets. LAMP-LFB reliably exhibited Cohen's kappa coefficient of 0.832 (95% CI, 0.721–0.943) showing a very good agreement between test and standard culture results as well as a high accuracy test determining by ROC analysis. However, two false-positive LAMP-LFB results apparently occurred, possibly due to the existence of sub-lethally damaged *C*. *perfringens* cells that could not be cultured under the culture conditions in this study. Meanwhile, six false-negatives were reported, possibly due to the fact that the samples might contain the bacterial cells below the minimum detection limit of the LAMP-LFB. On the other hand, false-negative results may occur from LAMP amplification failure caused by the presence of inhibitory substances in food samples [8, 9], especially in chili paste. It is possible that our DNA preparation protocols may still contain sample-borne LAMP inhibitors. These evidences indicated that the efficiency of DNA extraction is important, and our results agreed with a previous report that some inhibitors of LAMP reactions may not be removed by boiling method [8, 9]. To eliminate possible false-negative results, hence, the optimization of DNA extraction protocols may be warranted to further improve the outcome.

Interestingly, our data demonstrated that 41 of 70 chili paste samples (58.5%) and 3 of 20 cured meat samples (15.0%) were contaminated with *C*. *perfringens* according to standard culture and LAMP-LFB results. The high contamination of *C*. *perfringens* was found in chili paste from both fresh and supermarkets, indicating that the production process of chili paste lacked sanitation and hygiene conditions. This study supported the previous findings that powdered and dried chili, common ingredients used in chili paste, were highly contaminated with *C*. *perfringens* [5]. To prevent or minimize the risk of *C*. *perfringens* contamination, the good hygiene practice in the handling and preparation of chili paste should be considered and compliance by selecting fresh ingredient including raw meat and natural herbs in order to gain the highest hygiene and safety consumption with the requirement under the microbiological standard. Therefore, these findings support the use of LAMP-LFB assay developed in the current study for improving laboratory detection and the rapidity of results in microbiological quality control of chili paste.

In conclusion, our novel LAMP-LFB assay coupled with enrichment culture step represents the rapid, specific, sensitive and cost-effective detection for *C*. *perfringens* in varieties of food samples. The advantages of this assay include a naked-eye observation of the results, easy to perform with no requirement for special equipment, thus suitable for routine detection, testing and monitoring by food industries to rapidly detect *C*. *perfringens* in the samples. In addition, it also has potential for performance in facilities in developing countries, where resources are limited. Hence, this is the first report applying the novel LAMP-LFB assay for *C*. *perfringens* detection in food samples.

## Supporting information

S1 Fig Schematic illustration of LAMP-LFB process for detection of *C*. *perfringens*. (PDF)

S2 Fig The specificity of LAMP products for detecting different strains of *C*. *perfringens*. Twenty nanograms each of DNA templates were used in LAMP and subjected to (A) LFB in comparison to (B) 1.5% agarose gel electrophoresis. C and T indicate the control and test lines, respectively. A positive result displayed bands at both C and T, while a negative result showed one band at the C line. M: 2-log DNA ladder. 1–36: *C*. *perfringens* isolates (PF-1, 11, 17, 21, 26, 36, 38, 41, 44, 46, 47, 54, 58, 59, 63, 74, 76, 78, 80, 83, 93, 104, 132, 139, 147, 162, 176, 178, 179, 207, 208, 215, 242, 244, 254 and 257).
(PDF)

S3 Fig The LOD of LAMP-LFB using 10-fold serial dilutions of purified genomic DNA from *C*. *perfringens*. (PDF)

S4 Fig The LOD of LAMP-LFB and commercial real-time PCR kit using gravy sauce spiked with *C*. *perfringens*. The LOD was compared between LAMP-LFB (left) and commercial real-time PCR kit (right), enriched at 37°C for 0 h (A) and 16 h (B).
(PDF)

S5 Fig The LOD of LAMP-LFB and commercial real-time PCR kit using bologna spiked with *C*. *perfringens*. The LOD was compared between LAMP-LFB (left) and commercial real-time PCR kit (right), enriched at 37°C for 0 h (A) and 16 h (B).
(PDF)

S6 Fig Receiver operating curve analysis for assessing the detection accuracy of LAMP-LFB assay. The green line displayed the diagonal reference line, while the blue line displayed ROC plot between true positive rate (sensitivity) and false positive rate (1-specificity).
(PDF)

S1 Table Bacterial strains used in this study. (PDF)

S2 Table Demonstration of source of food samples used in this study. (XLSX)

S3 Table Detection of *C*. *perfringens* in food samples by culture-based method and LAMP-LFB assay. (PDF)

S4 Table Detection performance of LAMP-LFB assay for *C*. *perfringens* detection compared with culture-based method in naturally contaminated food samples. (PDF)

S1 File Original photographs of Figs 1B and S2B. (PDF)
