# Supplementary figures and images for: Rapid detection of Clostridium perfringens in food by loop-mediated isothermal amplification combined with a lateral flow biosensor
Source: PLoS One. 2021 Jan 7;16(1):e0245144. doi: 10.1371/journal.pone.0245144 (PMC7790239; doi:10.1371/journal.pone.0245144)

**S1 Fig.**

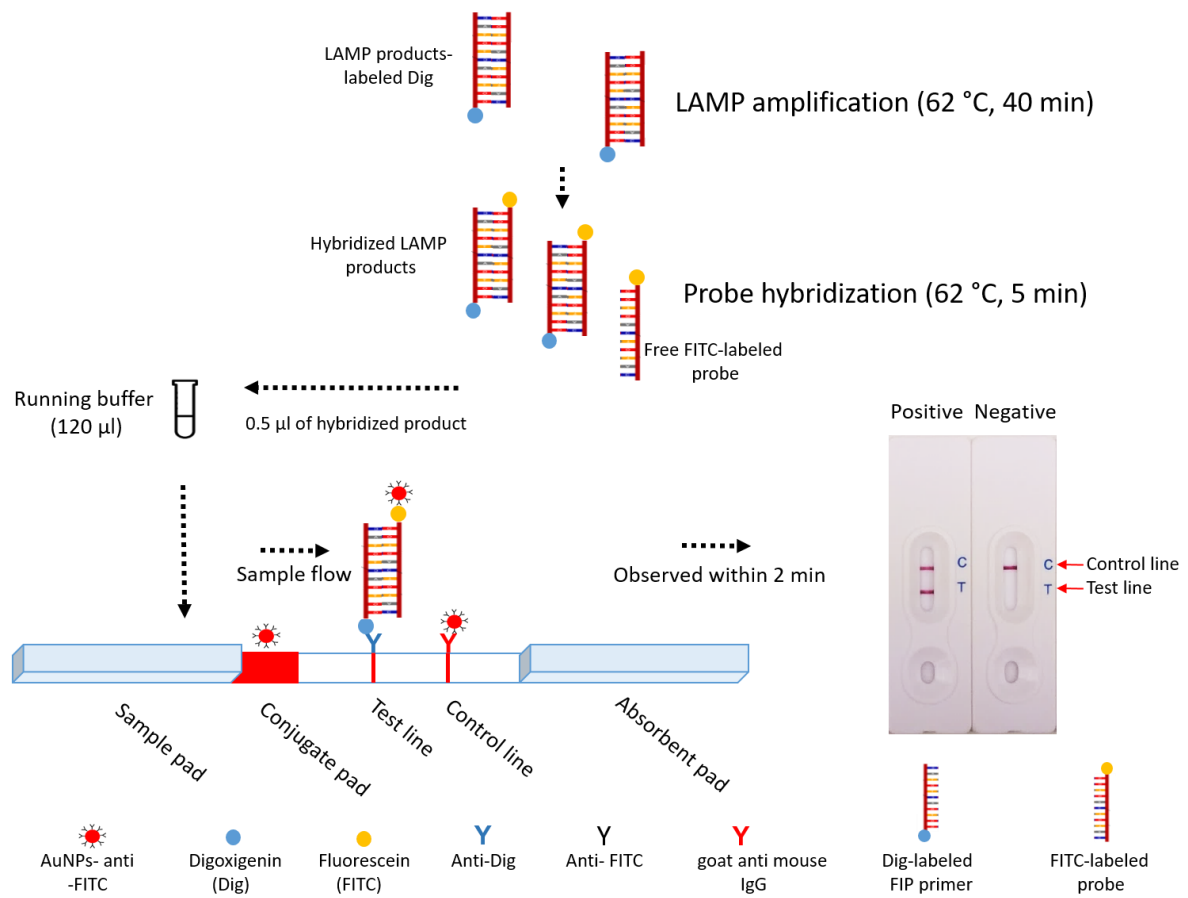

**S1 Fig. Schematic illustration of LAMP-LFB process for detection of *C. perfringens*.**

Supplement: S1 Fig — (PDF) [file pone.0245144.s001.pdf]
