# Supplementary material for: Rapid detection of Clostridium perfringens in food by loop-mediated isothermal amplification combined with a lateral flow biosensor
Source: PLoS One. 2021 Jan 7;16(1):e0245144. doi: 10.1371/journal.pone.0245144 (PMC7790239; doi:10.1371/journal.pone.0245144)
Supplement: S2 Fig — Twenty nanograms each of DNA templates were used in LAMP and subjected to (A) LFB in comparison to (B) 1.5% agarose gel electrophoresis. C and T indicate the control and test lines, respectively. A positive result displayed bands at both C and T, while a negative result showed one band at the C line. M: 2-log DNA ladder. 1–36: C. perfringens isolates (PF-1, 11, 17, 21, 26, 36, 38, 41, 44, 46, 47, 54, 58, 59, 63, 74, 76, 78, 80, 83, 93, 104, 132, 139, 147, 162, 176, 178, 179, 207, 208, 215, 242, 244, 254 and 257). (PDF) [file pone.0245144.s002.pdf]

S2 Fig.

(A)

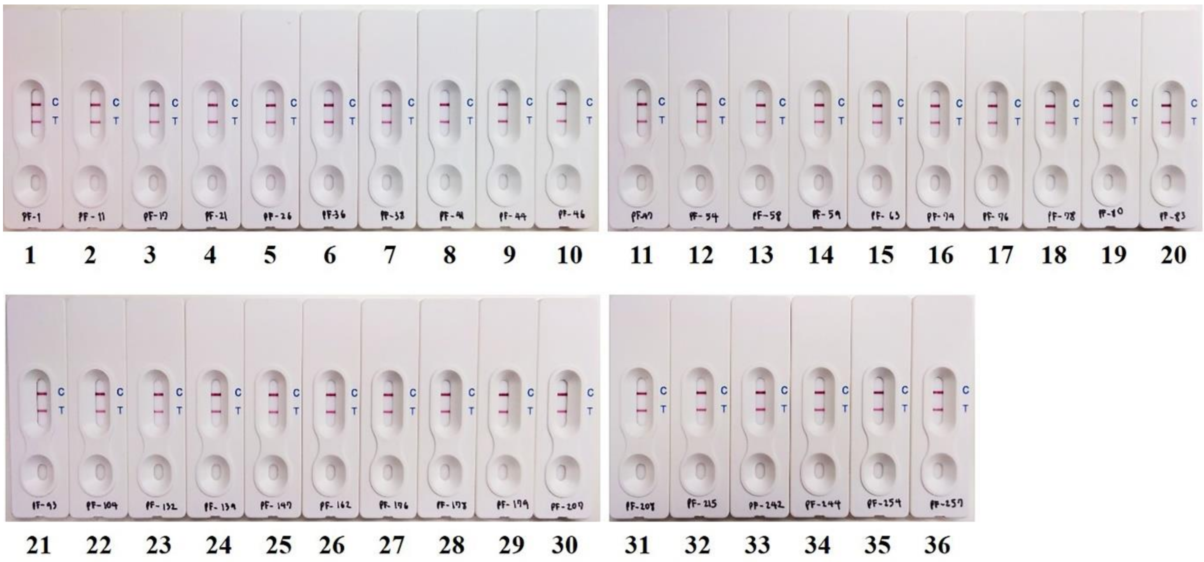

(B)

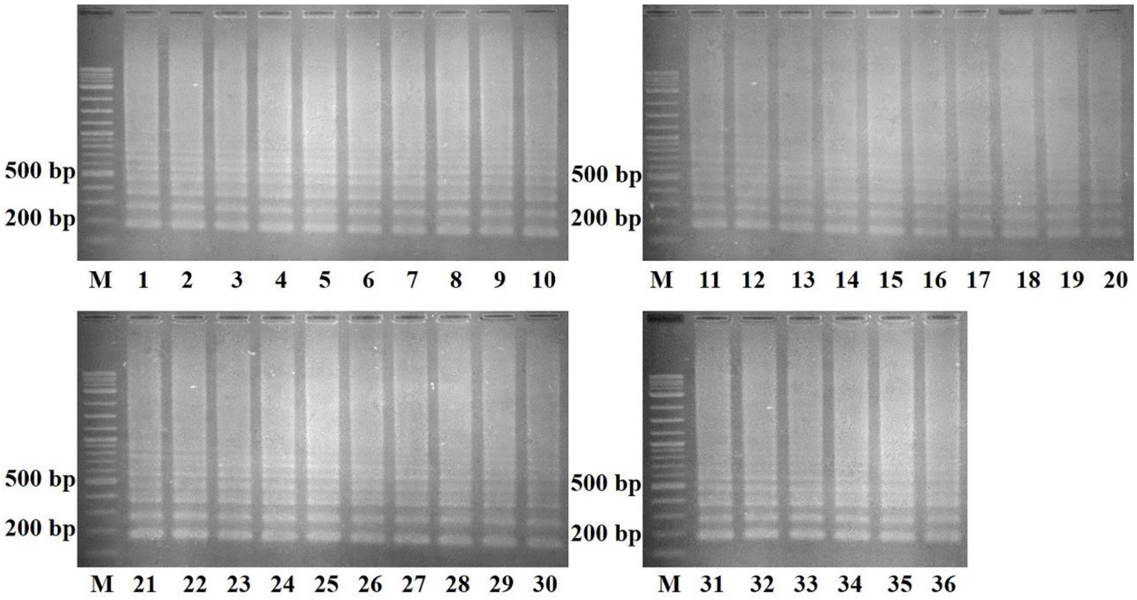

S2 Fig. The specificity of LAMP products for detecting different strains of *C. perfringens*. Twenty nanograms each of DNA templates were used in LAMP and subjected to (A) LFB in comparison to (B) 1.5% agarose gel electrophoresis. C and T indicate the control and test lines, respectively. A positive result displayed bands at both C and T, while a negative result showed one band at the C line. M: 2-log DNA ladder. 1-36: *C. perfringens* isolates (PF-1, 11, 17, 21, 26, 36, 38, 41, 44, 46, 47, 54, 58, 59, 63, 74, 76, 78, 80, 83, 93, 104, 132, 139, 147, 162, 176, 178, 179, 207, 208, 215, 242, 244, 254 and 257).
