# Supplementary material for: Rapid detection of Clostridium perfringens in food by loop-mediated isothermal amplification combined with a lateral flow biosensor
Source: PLoS One. 2021 Jan 7;16(1):e0245144. doi: 10.1371/journal.pone.0245144 (PMC7790239; doi:10.1371/journal.pone.0245144)
Supplement: S3 Fig — (PDF) [file pone.0245144.s003.pdf]

S3 Fig.

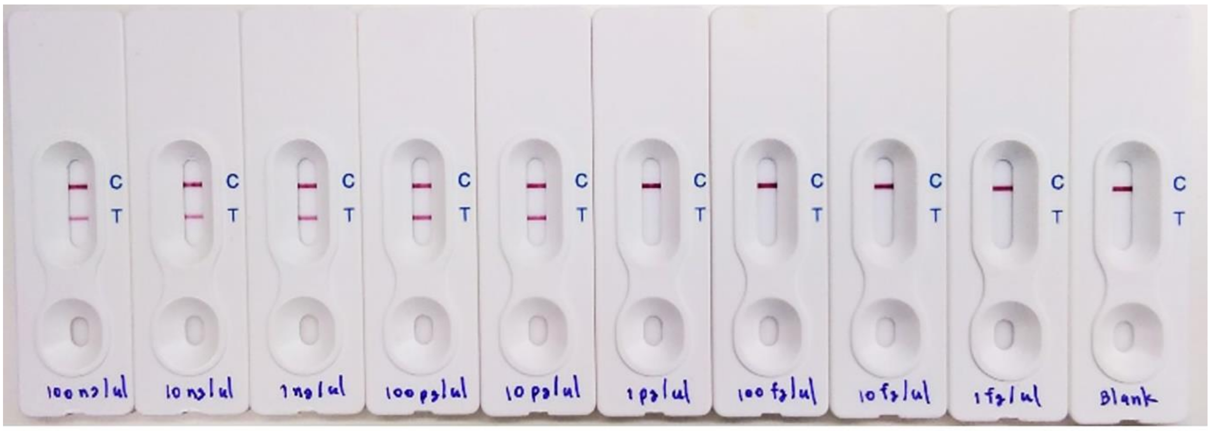

S3 Fig. The LOD of LAMP-LFB using 10-fold serial dilutions of purified genomic DNA from *C. perfringens*.
