# Supplementary material for: Rapid detection of Clostridium perfringens in food by loop-mediated isothermal amplification combined with a lateral flow biosensor
Source: PLoS One. 2021 Jan 7;16(1):e0245144. doi: 10.1371/journal.pone.0245144 (PMC7790239; doi:10.1371/journal.pone.0245144)
Supplement: S4 Fig — The LOD was compared between LAMP-LFB (left) and commercial real-time PCR kit (right), enriched at 37°C for 0 h (A) and 16 h (B). (PDF) [file pone.0245144.s004.pdf]

S4 Fig.

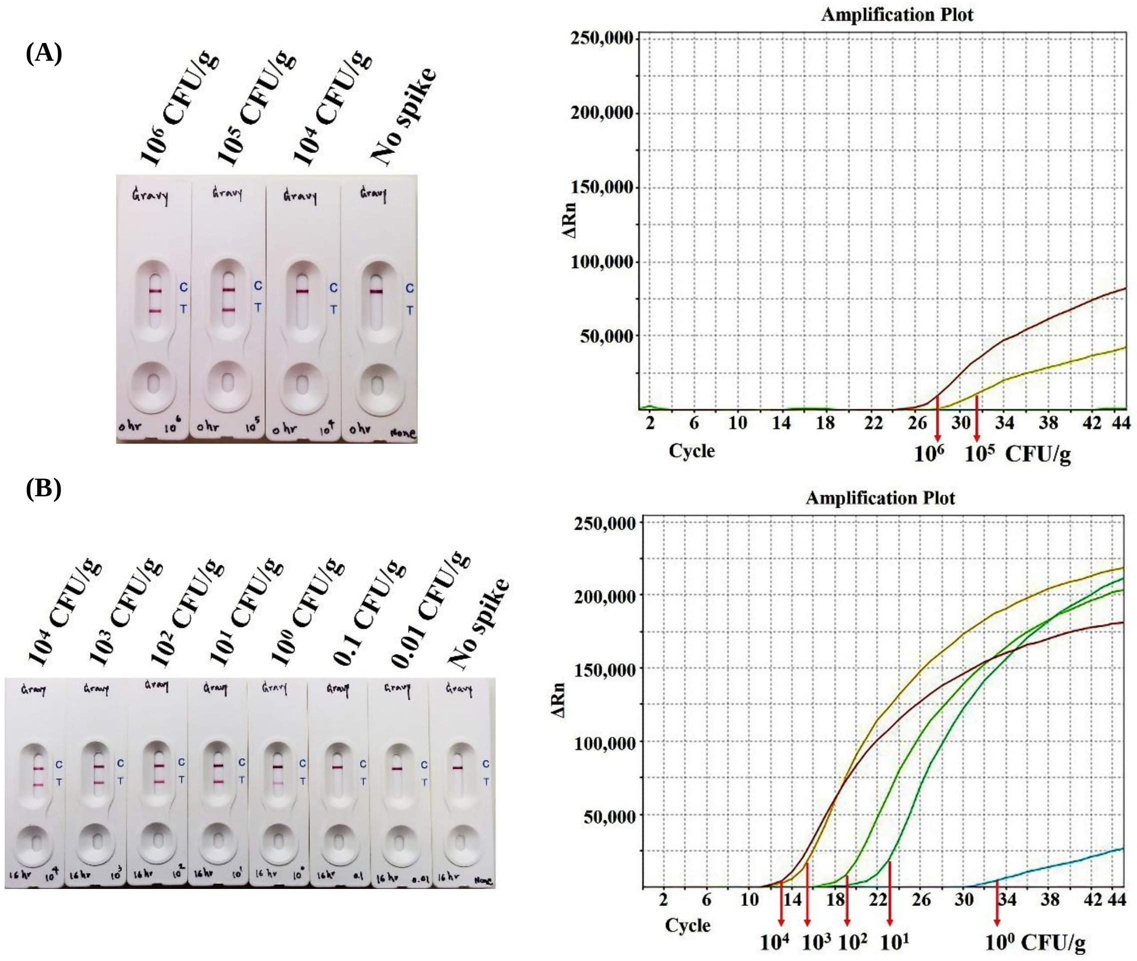

S4 Fig. The LOD of LAMP-LFB and commercial real-time PCR kit using gravy sauce spiked with *C. perfringens*. The LOD was compared between LAMP-LFB (left) and commercial real-time PCR kit (right), enriched at 37 °C for 0 h (A) and 16 h (B).
