# Supplementary material for: Rapid detection of Clostridium perfringens in food by loop-mediated isothermal amplification combined with a lateral flow biosensor
Source: PLoS One. 2021 Jan 7;16(1):e0245144. doi: 10.1371/journal.pone.0245144 (PMC7790239; doi:10.1371/journal.pone.0245144)
Supplement: S6 Fig — The green line displayed the diagonal reference line, while the blue line displayed ROC plot between true positive rate (sensitivity) and false positive rate (1-specificity). (PDF) [file pone.0245144.s006.pdf]

S6 Fig.

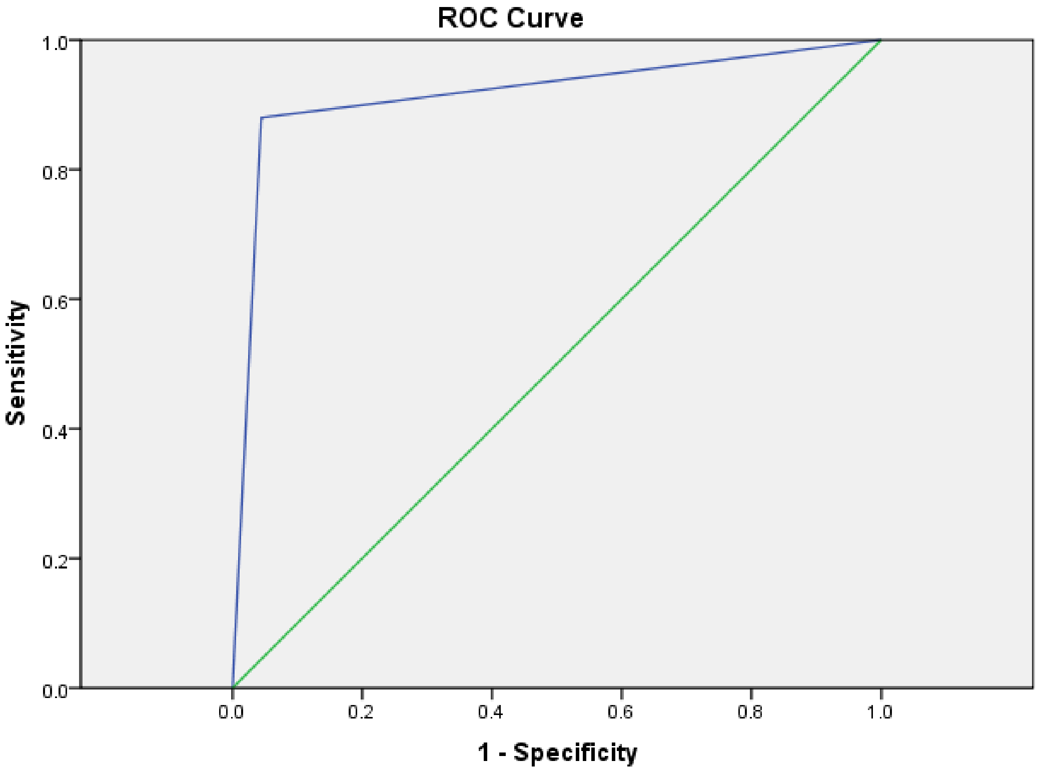

S6 Fig. Receiver operating curve analysis for assessing the detection accuracy of LAMP-LFB assay. The green line displayed the diagonal reference line, while the blue line displayed ROC plot between true positive rate (sensitivity) and false positive rate (1-specificity).
