# Supplementary material for: Rapid detection of Clostridium perfringens in food by loop-mediated isothermal amplification combined with a lateral flow biosensor
Source: PLoS One. 2021 Jan 7;16(1):e0245144. doi: 10.1371/journal.pone.0245144 (PMC7790239; doi:10.1371/journal.pone.0245144)
Supplement: S1 Table — (PDF) [file pone.0245144.s007.pdf]

S1 Table. Bacterial strains used in this study.

Bacterial species	Strains	Results	
		LAMP*	LAMP-LFB
Clostridium perfringens (type A)	DMST 16637	+	+
Clostridium perfringens (type B)	ATCC 3626	+	+
Clostridium perfringens (type C)	Ue 2130/06	+	+
Clostridium perfringens (type E)	NCTC 8084	+	+
Clostridium perfringens	PF-1	+	+
Clostridium perfringens	PF-11	+	+
Clostridium perfringens	PF-17	+	+
Clostridium perfringens	PF-21	+	+
Clostridium perfringens	PF-26	+	+
Clostridium perfringens	PF-36	+	+
Clostridium perfringens	PF-38	+	+
Clostridium perfringens	PF-41	+	+
Clostridium perfringens	PF-44	+	+
Clostridium perfringens	PF-46	+	+
Clostridium perfringens	PF-47	+	+
Clostridium perfringens	PF-54	+	+
Clostridium perfringens	PF-58	+	+
Clostridium perfringens	PF-59	+	+
Clostridium perfringens	PF-63	+	+
Clostridium perfringens	PF-74	+	+
Clostridium perfringens	PF-76	+	+
Clostridium perfringens	PF-78	+	+
Clostridium perfringens	PF-80	+	+
Clostridium perfringens	PF-83	+	+
Clostridium perfringens	PF-93	+	+
Clostridium perfringens	PF-104	+	+
Clostridium perfringens	PF-132	+	+
Clostridium perfringens	PF-139	+	+
Clostridium perfringens	PF-147	+	+
Clostridium perfringens	PF-162	+	+
Clostridium perfringens	PF-176	+	+
Clostridium perfringens	PF-178	+	+
Clostridium perfringens	PF-179	+	+
Clostridium perfringens	PF-207	+	+
Clostridium perfringens	PF-208	+	+
Clostridium perfringens	PF-215	+	+

S1 Table. (continued)

Bacterial species	Strains	Results	
		LAMP*	LAMP-LFB
Clostridium perfringens	PF-242	+	+
Clostridium perfringens	PF-244	+	+
Clostridium perfringens	PF-254	+	+
Clostridium perfringens	PF-257	+	+
Clostridium botulinum	NTCT 0727	-	-
Clostridium botulinum	NTCT 0751	-	-
Clostridium botulinum	NTCT 11219	-	-
Clostridium difficile	NIH 2	-	-
Clostridium difficile	R20291	-	-
Clostridium difficile	H37	-	-
Bacillus cereus	ATCC 14579	-	-
Campylobacter coli	DMST 18034	-	-
Campylobacter jejuni	DMST 15190	-	-
Escherichia coli	DMST 703	-	-
Listeria monocytogenes	DMST 17303	-	-
Salmonella Abony	DMST 21863	-	-
Salmonella Bangkok	DMST 7121	-	-
Salmonella Derby	DMST 8535	-	-
Salmonella Enteritidis	DMST 15676	-	-
Salmonella Hvittingfoss	DMST 15681	-	-
Salmonella Paratyphi B	DMST 28118	-	-
Salmonella Senftenberg	DMST 17013	-	-
Salmonella Typhi	DMST 22842	-	-
Salmonella Typhimurium	ATCC 23566	-	-
Salmonella Wandsworth	DMST 19204	-	-
Salmonella Waycross	DMST 19205	-	-
Shigella boydii	DMST 30245	-	-
Staphylococcus aureus	ATCC 25923	-	-
Staphylococcus aureus	DMST 8013	-	-
Staphylococcus aureus	DMST 4745	-	-
Staphylococcus epidermidis	DMST 15505	-	-
Staphylococcus haemolyticus	DMST 15511	-	-
Staphylococcus sacharolyticus	DMST 15512	-	-
Streptococcus pyogenes	DMST 4369	-	-
Streptococcus pneumoniae	DMST 7945	-	-
Streptococcus suis	DMST 18783	-	-

S1 Table. (continued)

Bacterial species	Strains	Results	
		LAMP*	LAMP-LFB
Vibio cholera	DMST 2873	-	-
Vibrio vulnificus	DMST 21245	-	-
Yersinia enterocolitica	DMST 8012	-	-

ATCC, American Type Culture Collection

DMST, Department of Medical Sciences Thailand

NCTC, National Collection of Type Cultures

PF, *C. perfringens* isolates

(‘+’, The assay is detected; ‘-’, The assay is not detected)

* 1.5% agarose gel electrophoresis applied for LAMP assay, while LFB applied for visual detection of *C. perfringens* - LAMP products.
