# Supplementary material for: Rapid detection of Clostridium perfringens in food by loop-mediated isothermal amplification combined with a lateral flow biosensor
Source: PLoS One. 2021 Jan 7;16(1):e0245144. doi: 10.1371/journal.pone.0245144 (PMC7790239; doi:10.1371/journal.pone.0245144)
Supplement: S3 Table — (PDF) [file pone.0245144.s009.pdf]

S3 Table. Detection of *C. perfringens* in food samples by culture-based method and LAMP-LFB assay.

Type of sample	Total of No.	Culture-based method		LAMP-LFB	
		No. of positive	No. of negative	No. of positive	No. of negative
Fresh markets					
Chili paste	35	27	8	22	13
Cured meat	10	2	8	2	8
Supermarket					
Chili paste	35	19	16	20	15
Cured meat	10	2	8	2	8
Gravy sauce	5	0	5	0	5
Total	95	50	45	46	49
