# Supplementary material for: Rapid detection of Clostridium perfringens in food by loop-mediated isothermal amplification combined with a lateral flow biosensor
Source: PLoS One. 2021 Jan 7;16(1):e0245144. doi: 10.1371/journal.pone.0245144 (PMC7790239; doi:10.1371/journal.pone.0245144)
Supplement: S4 Table — (PDF) [file pone.0245144.s010.pdf]

S4 Table. Detection performance of LAMP-LFB assay for *C. perfringens* detection compared with culture-based method in naturally contaminated food samples.

Method	Culture-based method				SE (%)	SP (%)	κ (%)
	Positive		Negative		(95% CI)	(95% CI)	(95% CI)
LAMP-LFB							
Positive	44	(TP)	2	(FP)	88.0	95.5	0.832
Negative	6	(FN)	43	(TN)	(75.6-95.4)	(84.8-99.4)	(0.721-0.943)

TP: True positive; TN: True negative; FP: False positive; FN: False negative

Sensitivity (SE) = (TP/TP+FN) ×100

Specificity (SP) = (TN/TN+FP) ×100

Cohen's kappa coefficient (κ) = 2× [(TP×TN) - (FN×FP)] / [(TP+FP) × (FP+TN)] + [(TP+FN) × (FN+TN)]

The κ ranged from 0-1, which defined as follow: ≤0.20 poor agreement, $0.21 < κ \leq 0.40$ fair agreement, $0.41 < κ \leq 0.60$ moderate agreement, $0.61 < κ \leq 0.80$ good agreement and ≥0.81 very good agreement
