# Supplementary material for: Rapid detection of Clostridium perfringens in food by loop-mediated isothermal amplification combined with a lateral flow biosensor
Source: PLoS One. 2021 Jan 7;16(1):e0245144. doi: 10.1371/journal.pone.0245144 (PMC7790239; doi:10.1371/journal.pone.0245144)
Supplement: S1 File — (PDF) [file pone.0245144.s011.pdf]

Original photographs of Fig 1B.

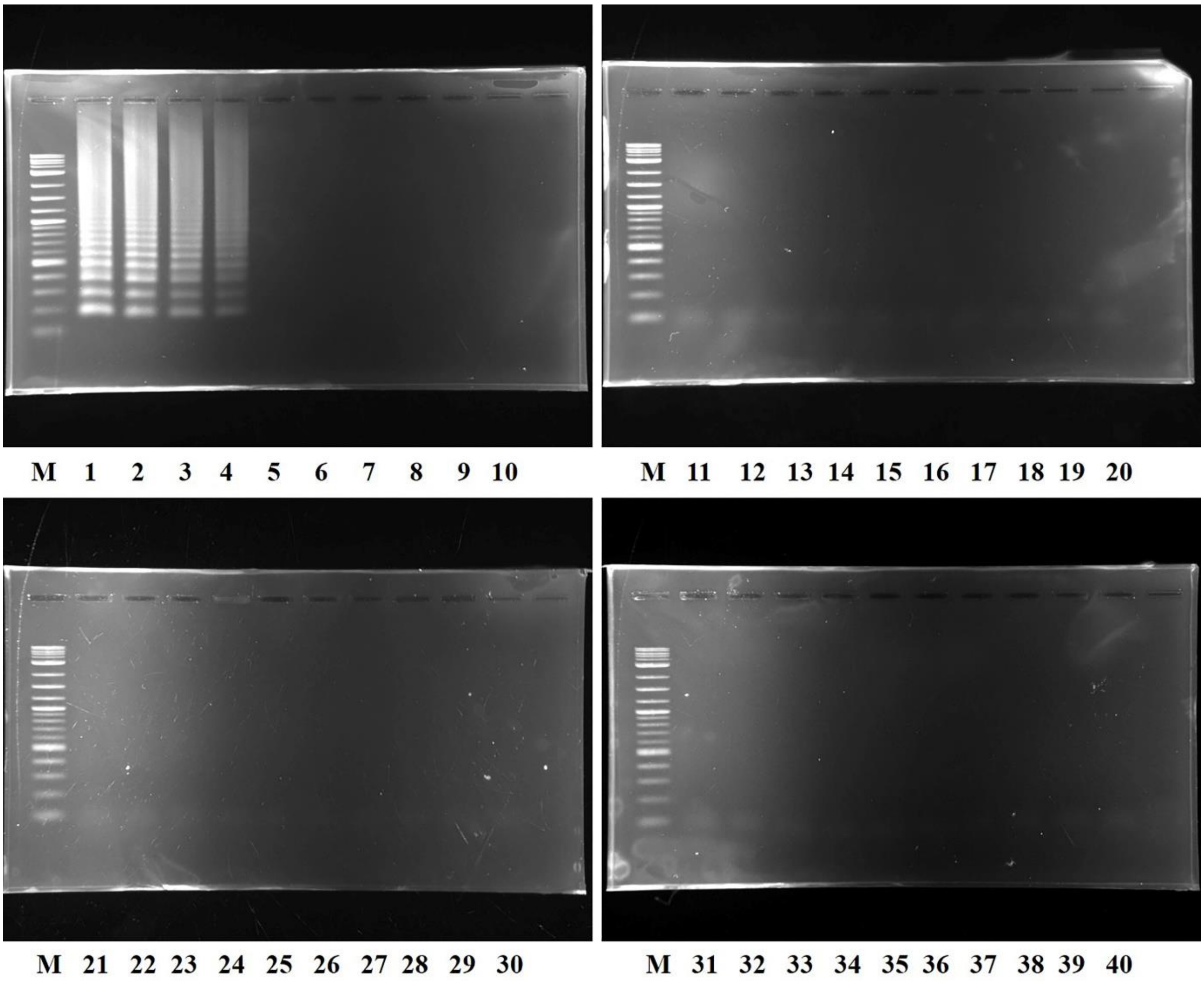

The DNA marker is 2-log DNA ladder (0.1-10.0 kb, No. N3200S) (New England Biolabs Inc., USA). The photo was taken with transilluminator (Syngene, USA).

Original photographs of S2B Fig.

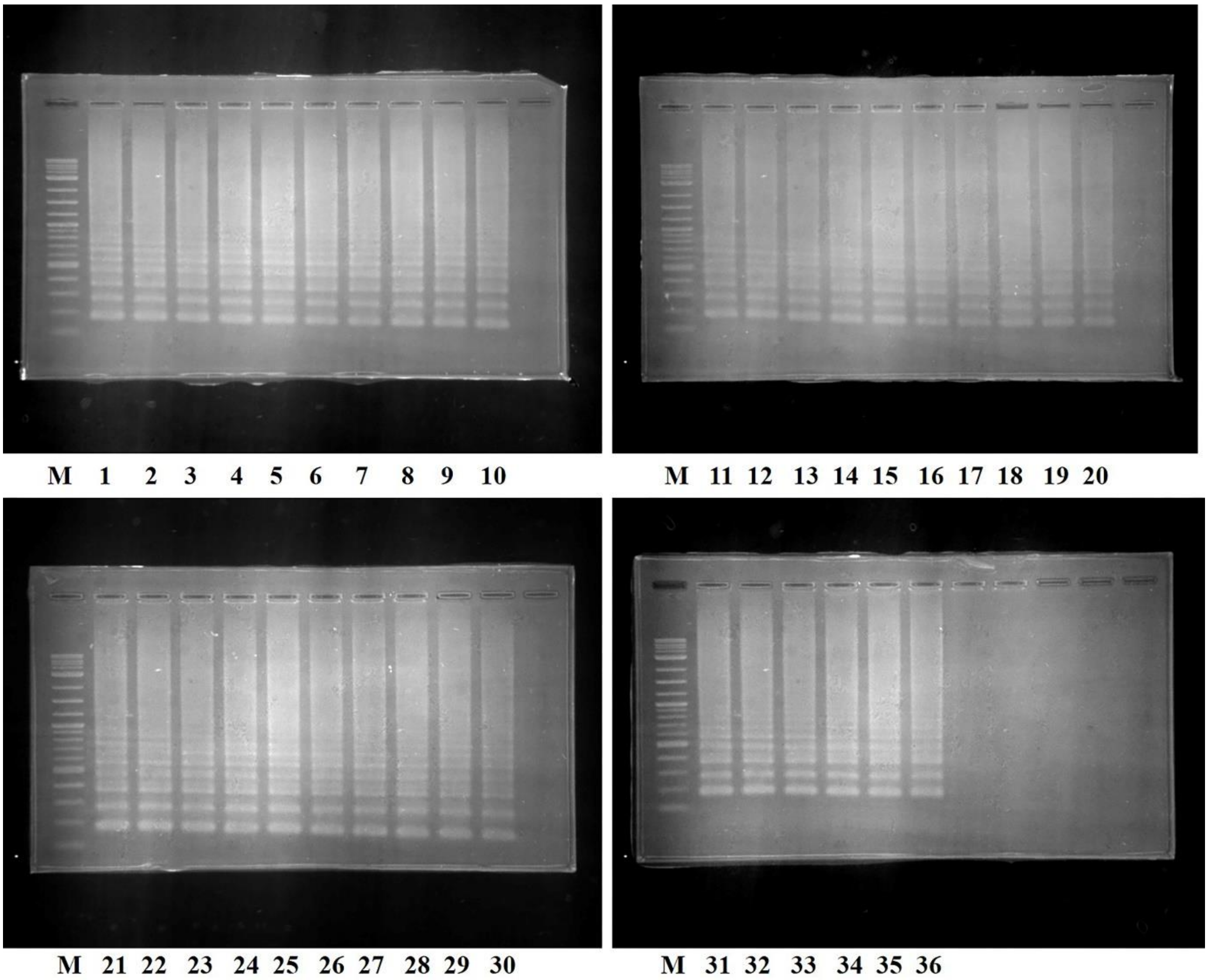

The DNA marker is 2-log DNA ladder (0.1-10.0 kb, No. N3200S) (New England Biolabs Inc., USA). The photo was taken with transilluminator (Syngene, USA).
